# Cannabinoids as Key Regulators of Inflammasome Signaling: A Current Perspective

**DOI:** 10.3389/fimmu.2020.613613

**Published:** 2021-01-28

**Authors:** Santosh V. Suryavanshi, Igor Kovalchuk, Olga Kovalchuk

**Affiliations:** Department of Biological Sciences, University of Lethbridge, Lethbridge, AB, Canada

**Keywords:** inflammasome, pro-inflammatory cytokines, inflammasome signaling, cannabidiol, Delta-9 tetrahydrocannabinol, cannabinoids

## Abstract

Inflammasomes are cytoplasmic inflammatory signaling protein complexes that detect microbial materials, sterile inflammatory insults, and certain host-derived elements. Inflammasomes, once activated, promote caspase-1–mediated maturation and secretion of pro-inflammatory cytokines, interleukin (IL)-1β and IL-18, leading to pyroptosis. Current advances in inflammasome research support their involvement in the development of chronic inflammatory disorders in contrast to their role in regulating innate immunity. Cannabis (marijuana) is a natural product obtained from the *Cannabis sativa* plant, and pharmacologically active ingredients of the plant are referred to as cannabinoids. Cannabinoids and cannabis extracts have recently emerged as promising novel drugs for chronic medical conditions. Growing evidence indicates the potent anti-inflammatory potential of cannabinoids, especially Δ^9^-tetrahydrocannabinol (Δ^9^-THC), cannabidiol (CBD), and synthetic cannabinoids; however, the mechanisms remain unclear. Several attempts have been made to decipher the role of cannabinoids in modulating inflammasome signaling in the etiology of chronic inflammatory diseases. In this review, we discuss recently published evidence on the effect of cannabinoids on inflammasome signaling. We also discuss the contribution of various cannabinoids in human diseases concerning inflammasome regulation. Lastly, in the milieu of coronavirus disease-2019 (COVID-19) pandemic, we confer available evidence linking inflammasome activation to the pathophysiology of COVID-19 suggesting overall, the importance of cannabinoids as possible drugs to target inflammasome activation in or to support the treatment of a variety of human disorders including COVID-19.

## Introduction

Animals, from lower vertebrates, such as hagfish, to higher ones, such as mammals, use innate, adaptive immune responses to protect themselves from external pathogens and injuries (1, 2). An innate immune response is the first event upon any hazard where external microbes are sensed by a group of diverse germline-encoded receptors. These receptors-pattern recognition receptors (PRRs)-recognize conserved microbial structures called pathogen-associated molecular patterns (PAMPs) (3). Innate immune cells can also mount an infection-independent immune response by sensing endogenous substances released from host tissue damage: damage (or danger)-associated molecular patterns (DAMPs) (4). This type of DAMP-mediated inflammatory response is often called “sterile inflammation” due to no pathogen involvement (5). Overall, both PAMPs and DAMPs can stimulate an initial immune response by activating different types of PRRs, including toll-like receptors (TLRs), nucleotide-binding domain (NBD) and leucine-rich-repeat-(LRR)-containing or simply nucleotide-binding and oligomerization domain (NOD)-like receptors (NLRs), retinoic acid-inducible gene I (RIG-I)-like receptors (RLRs), C-type lectin receptors (CLRs), and several cytosolic DNA sensor-like receptors absent in melanoma 2 (AIM2)-like receptors (ALRs) (4, 6). These PRRs are expressed by immune cells (macrophages, dendritic cells, etc.) and non-immune cells (endothelial cells, fibroblasts, etc.) and are present in various subcellular compartments (3, 4). Among all PRRs, the NLR family is the most extensively described in the literature due to numerous known sterile and pathogenic activators (7). NLRs and ALRs form a multimeric complex known as the “inflammasome” following the detection of respective PAMPs and/or DAMPs.

TLR-induced priming is often required to activate and assemble the inflammasome (8). Once formed and activated at the cellular level, canonical inflammasomes activate caspase-1 by autoproteolysis, resulting in proteolytic maturation of IL-1β and IL-18, which in turn leads to respective cell death by pyroptosis. Non-canonical inflammasome activation by bacterial pathogens leads to caspase-11 activation and the subsequent hyper-activation of innate immunity in mice (9). Nevertheless, IL-1β and IL-18 are crucial pro-inflammatory cytokines implicated in a variety of human disorders, such as aging, lung cancer, cardiovascular diseases, gout, etc (10, 11). Besides pyroptosis, inflammasomes may be involved in eicosanoid synthesis, phagosome maturation, glycolysis, lipid metabolism, and autophagy in a cytokine- and pyroptosis-independent manner (12).

*Cannabis sativa* (*C. sativa*) has been cultivated for centuries around the world for many purposes, and it is the most frequently used illegal plant. “Marijuana” is the term used to describe cannabis varieties that contain more than 0.3% Δ^9^-THC by dry weight, while “hemp” is used for varieties with lower than 0.3% Δ^9^-THC. *C. sativa* is a versatile plant that provides food, feed, shelter, and medicine. Since ancient times, various cannabis preparations have been used in both traditional and professional medicine. Although cannabis can be beneficial in treating various human diseases (13), evidence-based medical conditions for which cannabis can be usefully prescribed are chronic pain, nausea and vomiting after chemotherapy, seizures in Lennox-Gastaut and Dravet syndrome, and spasticity (14, 15). On the other hand, a recent study suggests that newly prescribed cannabinoid use (either nabilone or dronabinol) among older adults with the chronic obstructive pulmonary disorder (COPD) was associated with higher rates of adverse events. Although further research is needed to confirm the same, the physicians should weigh benefits against risks while prescribing new cannabinoids to older COPD patients (16). At least 554 compounds, including 113 phytocannabinoids and 120 terpenes, have been identified in *C. sativa* (13). Terpenes in cannabis give the plant a characteristic odor based on percentages of various volatile aromatic compounds. Terpenes are believed to be partially responsible for the “entourage effect,” with minor cannabinoids and other molecules such as phenolic compounds having additional effects. To date, no clear evidence has emerged for the role of any molecule or their combination in the entourage effect. In fact, recent experiments have demonstrated that terpenes do not add to the activation of cannabinoid receptors triggered by cannabinoids (17, 18). It remains to be shown, however, whether they can contribute to the entourage effect through interaction with other receptors.

Several publications have demonstrated the potent anti-inflammatory effect of cannabinoids (19). Their mechanisms of action include activating cannabinoid and other receptors, inhibiting cytokines and cell proliferation, inducing apoptosis, and so on (19–21). Inflammation occurs when innate immune cells detect pathogens, injury, or danger signals *via* PRRs on cell membranes and in cytosols. Activated PRRs then form inflammasomes, triggering signaling cascades leading to the recruitment of leukocytes to the injury site (22). Under normal conditions, acute inflammatory events characterized by the influx of neutrophils at the injured tissue are crucial parts of innate immunity. However, dysregulated acute inflammation, sterile inflammation, and recurrent acute inflammatory insults result in chronic inflammation. Chronic inflammation has been implicated in the pathophysiology of a variety of diseases. Inflammasomes are activated during microbial invasion, tissue injury, and sterile inflammation, which all lead to cell death. Cell death can also result in the secretion of another round of inflammasome activators, such as uric acid and ATP, which both activate inflammasomes in a paracrine manner. These signaling cascades eventually give rise to chronic inflammatory disorders, such as cardiovascular disease, cancer, metabolic disorders, autoimmune disorders, and neurodegenerative disorders (23). Besides, recent developments in inflammasome research suggest that the anti-inflammatory action of cannabinoids is mediated in part by modulating inflammasome assembly and function. Hence, our goals in this review are to cover all published research on the action of cannabinoids on the inflammasome to propose the future therapeutic potential of cannabis in chronic inflammatory disorders.

## CANNABINOIDS Signaling

The first documented evidence of the medicinal use of *C. sativa* showed that its extracts were already in use around 5,000 years ago in ancient China to alleviate pain (24). Three types of cannabinoids exist: *endocannabinoids* produced by the human body; *phytocannabinoids* produced naturally by *C. sativa*; and *synthetic cannabinoids* synthesized under laboratory conditions. After the discovery of Δ^9^-THC (25), extensive research efforts were carried out to understand the pharmacological effects of cannabis. Eventually, two members of the G-protein coupled receptor (GPCR) family, the cannabinoid receptors CB1R and CB2R, were successfully cloned from rat cerebral cortex and rat spleen, respectively (26, 27). Many cannabinoids were demonstrated to bind these receptors, albeit with different efficiencies. Cannabinoids were also shown to bind to receptors other than CB1R and CB2R, as reviewed by our group elsewhere (28). All three types of cannabinoids exert their biological actions by binding to these receptors, and each cannabinoid may bind different combinations of receptors at a given time.

The endocannabinoid system (ECS) comprises of two endocannabinoids (anandamide and 2-arachidonoylglycerol), cannabinoid receptors (CB1 and CB2), and enzymes that metabolize endocannabinoids (29). Anandamide (AEA) (30) and 2-arachidonylglycerol (2-AG) (31) are the two most important endocannabinoids, although other arachidonic acid derivatives may produce similar effects (28). Both AEA and 2-AG are produced from postsynaptic terminals owing to increased intracellular Ca^2+^ influx (24, 32). Once produced, AEA and majorly 2-AG travel in a retrograde fashion due to their high lipophilicity to activate CB1 receptors in presynaptic terminals. The activation of CB1R inhibits neurotransmitter release *via* the reduction of Ca^2+^ inflow and inhibition of cyclic adenosine monophosphate (cAMP). However, AEA activates the intracellular transient receptor potential cation channel subfamily V member 1 (TRPV1) receptor, inhibits L-type Ca^2+^ channels, and inhibits 2-AG biosynthesis (24). The major role of ECS in the body is to maintain homeostasis, and it is involved in the regulation of a variety of processes, including immune, digestive, neurological, metabolic, and reproductive functions (29, 33). Variations in the ECS are pathophysiological and depend on cell and tissue type, age, and sex, and fluctuations in the function of ECS components are associated with the onset of many disorders, such as neurodegenerative, gastrointestinal, chronic inflammatory, cardiovascular, reproductive, circulatory, and metabolic disorders, including cancers (24, 34, 35).

The human CB1R and CB2R crystal structures reveal that both the intramembrane and the extracellular surface of the receptor play significant role in the ligand binding, depending on the type of ligand, unlike other lipid-stimulated GPCRs (36, 37). The extracellular-facing lid over intracellular binding pocket with its acidic residues facing outside disfavors interactions with lipophilic ligands in the extracellular space, which gain entrance to the binding pocket *via* an intramembrane “tunnel” (37). CB1 and CB2 receptors share only 44% of the homology of the protein sequence in humans, and CB1 receptors are highly conserved among mammals in contrast to CB2 (38). Overall, CB1R is the most extensively expressed GPCR in the central nervous system (CNS) and the peripheral nervous system (PNS), whereas CB2R is highly expressed in peripheral immune cells. However, the functional CB1Rs are also expressed in several non-neuronal peripheral tissues including heart, intestines, and liver (39). Additionally, the functional CB2 receptors are also expressed in microglia and resident macrophages in the CNS under neuroinflammatory conditions (40, 41). CB2R expression was confirmed in the neurons, as well, governing synaptic plasticity (42). Both receptors are coupled to the G_i/o_ family of G-proteins to inhibit cAMP production by reducing adenylyl cyclase activity, leading to lower protein kinase A (PKA) activity and the phosphorylation of mitogen-activated protein kinase (MAPK) activity (p38, c-Jun N-terminal kinase (JNK), and p42/44). They also can activate phosphatidylinositol-3-kinase-protein kinase B (PI3K-AKT), ceramide production, and the expression of various genes (24, 41, 43, 44). Interestingly, CB1 and CB2 receptors interact with G_s_ proteins as well to induce cAMP production under specific circumstances (45, 46). CB1 receptor activation specifically stimulates G-protein-gated inwardly rectifying potassium (GIRK) channels and inhibits voltage-gated (N-type) Ca^2+^ channels. Both receptors exhibit constitutive activity suggestive of G-protein activation in the absence of agonists (42). Lastly, CB1 and CB2 receptors also signal *via* β-arrestin and a few other biased, cell-, and ligand-specific cannabinoid receptor–mediated signal transduction mechanisms (47).

AEA is a highly specific partial agonist of CB1R with negligible or weak partial agonist activity at CB2R, whereas 2-AG is a full agonist at both CB1 and CB2 receptors. Δ^9^-THC has the highest affinity toward CB1R and CB2R among all phytocannabinoids (48). CBD is a weak antagonist at CB1 and an inverse agonist at CB2 receptors (49), although a meta-analysis study revealed that CBD mostly acts indirectly through other signaling pathways (50). CBD acts as an agonist at adenosine receptors and 5-HT_1A_ receptors, and it increases AEA levels to elicit TRPV1 channel activation (50). Other minor cannabinoids also bind to cannabinoid and other receptors, as reviewed by our group elsewhere (28). By modulating intracellular cAMP levels and thereby PKA activity, cannabinoids regulate the phosphorylation of a plethora of downstream proteins, resulting in major changes in cellular activities. MAPK activation is an important pathway by which cannabinoids regulate the expression of various genes. Changes in the extracellular and intracellular ions and activation of ion channels contribute to crucial downstream cellular effects (44). Due to the possibility of binding to different receptors at a given time, including CB1R and CB2R, along with different respective agonist/antagonist/inverse agonist potentials at those receptors, cannabinoids exhibit complexity in their mechanisms of action and downstream signaling transduction pathways.

## Cannabinoids in Inflammation

Cannabinoids and cannabinoid-like compounds have proven potent anti-inflammatory and immunomodulatory properties (19, 21, 35, 51, 52). In general, cannabinoids work by inducing apoptosis, preventing cell proliferation, reducing cytokine production, and enhancing T-regulatory cells (Tregs) to produce anti-inflammatory effects (19). Interestingly, cannabinoids may change the balance between the response involving T-helper 1 (Th-1) and Th-2 cells, inhibiting the expression of Th-1–induced cytokines and stimulating the expression of Th-2–induced cytokines (53). More than 350 patents have been filed on cannabinoids in the treatment of inflammation (54). Ajulemic acid (anabasum), a novel selective CB2R agonist, is currently undergoing phase II and phase III clinical trials owing to its potent anti-inflammatory effect on neutrophil migration in response to ultraviolet (UV)‐killed *E. coli*‐triggered dermal inflammation in humans. Notably, ajulemic acid removed the pathogenic bacteria that caused the inflammation and promoted the biosynthesis of special pro‐resolution lipid mediators to boost the body’s innate immunity (55, 56). The data from recently concluded RESOLVE-1 phase III trial of anabasum for the treatment of systemic sclerosis failed to provide any efficacy in the primary or secondary end points (Corbus Pharmaceuticals Press Release Sept 8, 2020). However, the additional post-hoc analyses released by Corbus recently showed that anabasum treatment was associated with a benefit in the lung function (forced vital capacity) in subjects on established background of immunosuppressant therapies (Corbus Pharmaceuticals Press Release Nov 9, 2020). Overall, anabasum was able to improve the lung function in patients with systemic sclerosis although was not effective in improving the actual end points of the clinical trial (NCT03398837).

CBD is the most abundant non-psychoactive cannabinoid of *C. sativa* and hence has been extensively studied for its anti-inflammatory properties. CBD is currently undergoing clinical trials for its effectiveness in schizophrenia (57), refractory epileptic encephalopathy (58), and tuberous sclerosis (59, 60). In addition to CB1, CB2, TRPV1, and adenosine receptors, the activation of GPR55, inhibition of fatty acid amide hydrolase (FAAH), stimulation of peroxisome proliferator-activated receptor-gamma (PPAR-γ), and heterodimerization of CB2/5HT_1A_ are also involved in mediating the anti-inflammatory effects of CBD (20, 61, 62). Subsequently, CBD was also found to extensively inhibit the production of pro-inflammatory cytokines, such as IL-1α, IL-1β, IL-6, and tissue necrosis factor α (TNF-α), etc., in pre-clinical *in vitro* and *in vivo* models of inflammation and cancer (20). The anti-inflammatory activity of CBD was paralleled by the modulation of downstream gene expression, reduction in lipid peroxidation, Ca^2+^ homeostasis, and reduction of oxidative stress (61, 63, 64). However, Δ^9^-THC mediates its anti-inflammatory actions mainly *via* CB2 receptor activation, decreased production of cytokines, inhibition of Th-1, promotion of Th-2 cells, induction of apoptosis, and downregulation of cell proliferation (35, 52). Cannabichromene (CBC) has been reported to inhibit the expression and activity of TRPV1-4 channels (65). Cannabigerol (CBG) has exhibited protective properties in a murine model of inflammatory bowel disease (IBD) by regulating cytokine (IL-1β, IL-10, and interferon-γ) levels and inhibiting inducible nitric oxide synthase (iNOS) expression (66). Cannabinol (CBN), like CBD and THC, is shown to inhibit pro-inflammatory cytokine production (67). Cannabidiolic acid (CBDA) has been demonstrated to be a selective inhibitor of cyclooxygenase-2 (COX-2), and it likely plays an essential role in the reduction of inflammation (68). Data on other minor cannabinoids are limited at this point.

## Inflammasome Signaling

**Figure 1:**
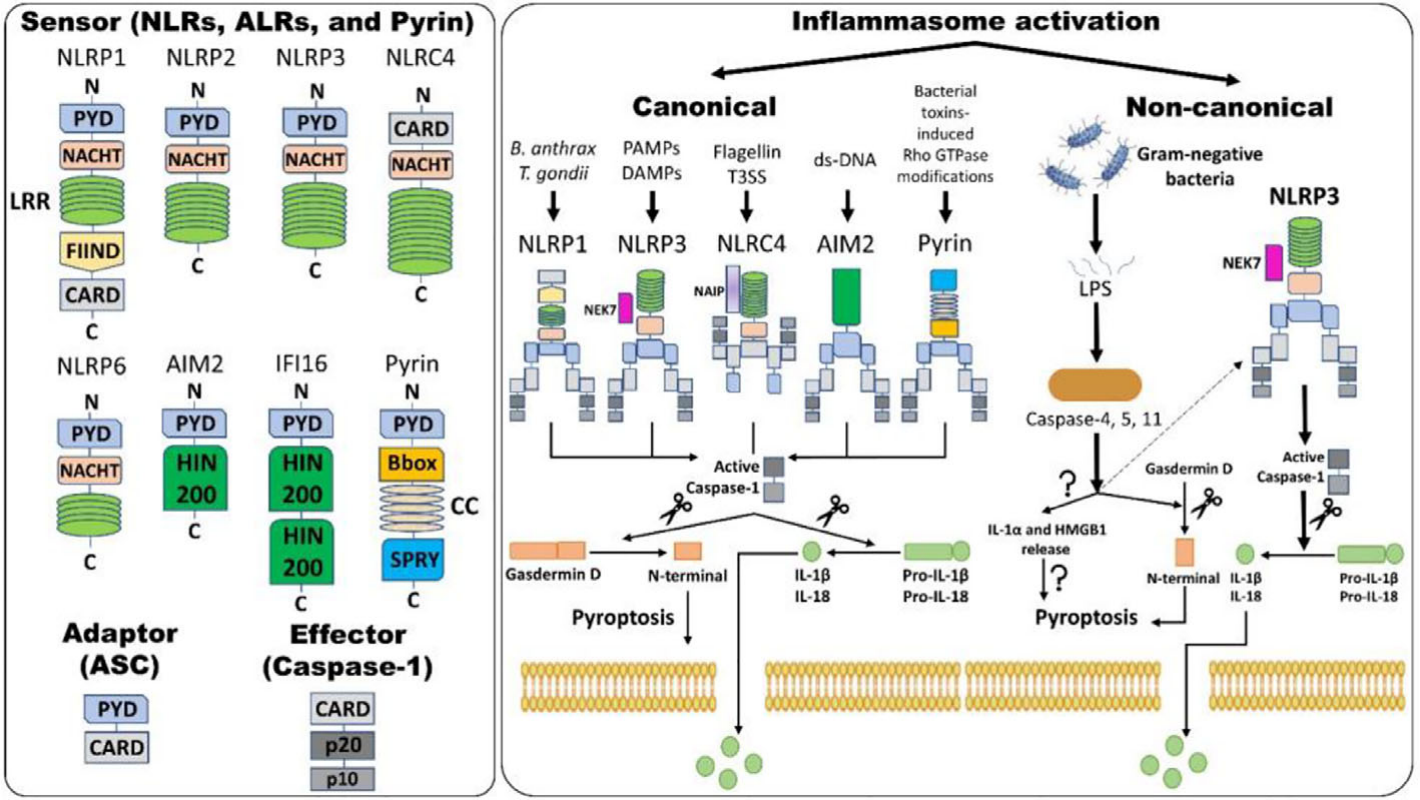
Inflammasome assembly and activation pathways. An inflammasome consists of three proteins: sensor, adaptor, and effector. Please note that NLRP3 and NLRC4 require NIMA (Never in Mitosis Gene A)-related Kinase 7 (NEK7) and NLR family apoptosis inhibitory protein (NAIP), respectively, for activation. Canonical inflammasome activation results in the formation of active caspase-1 (activation) by removing caspase activation and recruitment domain (CARD; processing). Active caspase-1 cleaves gasdermin D (GSDMD) and pro-interleukins into the N-terminal of GSDMD and mature interleukins, respectively. The N-terminal of GSDMD forms pores within the cell membrane, allowing mature IL-1β and IL-18 release along with changes in ion fluxes. Such caspase-1-dependent formation of plasma membrane pores releasing inflammatory intracellular materials resulting in cell lysis is termed pyroptosis. Alternatively, in non-canonical inflammasome activation, gram-negative bacteria release lipopolysaccharides (LPS) that activate caspase-4 and -5 in humans and caspase-11 in mice, which results in pyroptosis *via* several mechanisms. Firstly, GSDMD-mediated pyroptosis occurs as explained above and, secondly, activation of caspases leads to the release of IL-1α and high-mobility group box protein 1 (HMGB1) *via* unknown mechanisms, which results in pyroptosis. Caspase-4, -5, and -11 activation by LPS also indirectly activates the NLRP3 inflammasome, culminating in the maturation of IL-1β and IL-18 *via* activating caspase-1. Abbreviations: Nucleotide-binding domain (NBD) and leucine-rich-repeat-(LRR)-containing receptors (NLRs); Absent in melanoma 2 (AIM2)-like receptors (ALRs); pyrin domain (PYD); Caspase activation and recruitment domain (CARD); Nucleotide-binding and oligomerization or NAIP, CIITA, HET-E and TP1 (NACHT) domain; Apoptosis-associated speck-like protein containing CARD (ASC); Interferon-gamma inducible protein 16 (IFI-16); Function-to-find domain (FIIND); Coiled-coil (CC); Bacterial type III secretion system (T3SS); Hematopoietic interferon-inducible nuclear protein with 200 amino acids (HIN-200).

The “inflammasome” is the name given to the high molecular weight scaffold formed by an assembly of different proteins. This scaffold mostly consists of three parts (1): a sensor protein, (2) typically the adaptor protein ASC (an apoptosis-associated, speck-like protein containing a C-terminal caspase recruitment domain [CARD]), and (3) the effector protein caspase-1 (cysteine protease) (12, 69). Sensor proteins are six NLRs (NLRP1, NLRP3, NLRP6, NLRP7, NLRP12, and NLRC4) or two ALRs (AIM2 and interferon-gamma inducible protein 16 (IFI-16) or pyrin (PYD). The NLR family contains the central nucleotide-binding and oligomerization (NACHT) domain flanked by C-terminal leucine-rich repeats (LRRs) and N-terminal CARD or PYD domains. LRRs specifically govern the ligand sensing for each NLR and autoregulation, whereas CARD or PYD domains regulate the protein-protein interactions required for downstream signaling cascades. The NACHT domain is shared by all NLRs and regulates NLR activation *via* adenosine 5′-triphosphate (ATP)-induced oligomerization (69). ASC is required for certain PRRs, NLRP3, AIM2, and PYD to recruit caspase-1, and NLRs such as NLRP1 and NLRC4 contain CARD and hence directly recruit caspase-1. Although other PRRs might not need ASC for caspase-1 recruitment, downstream cytokine processing depends on ASC in the complex (70) (**Figure 1**).

**Figure 2:**
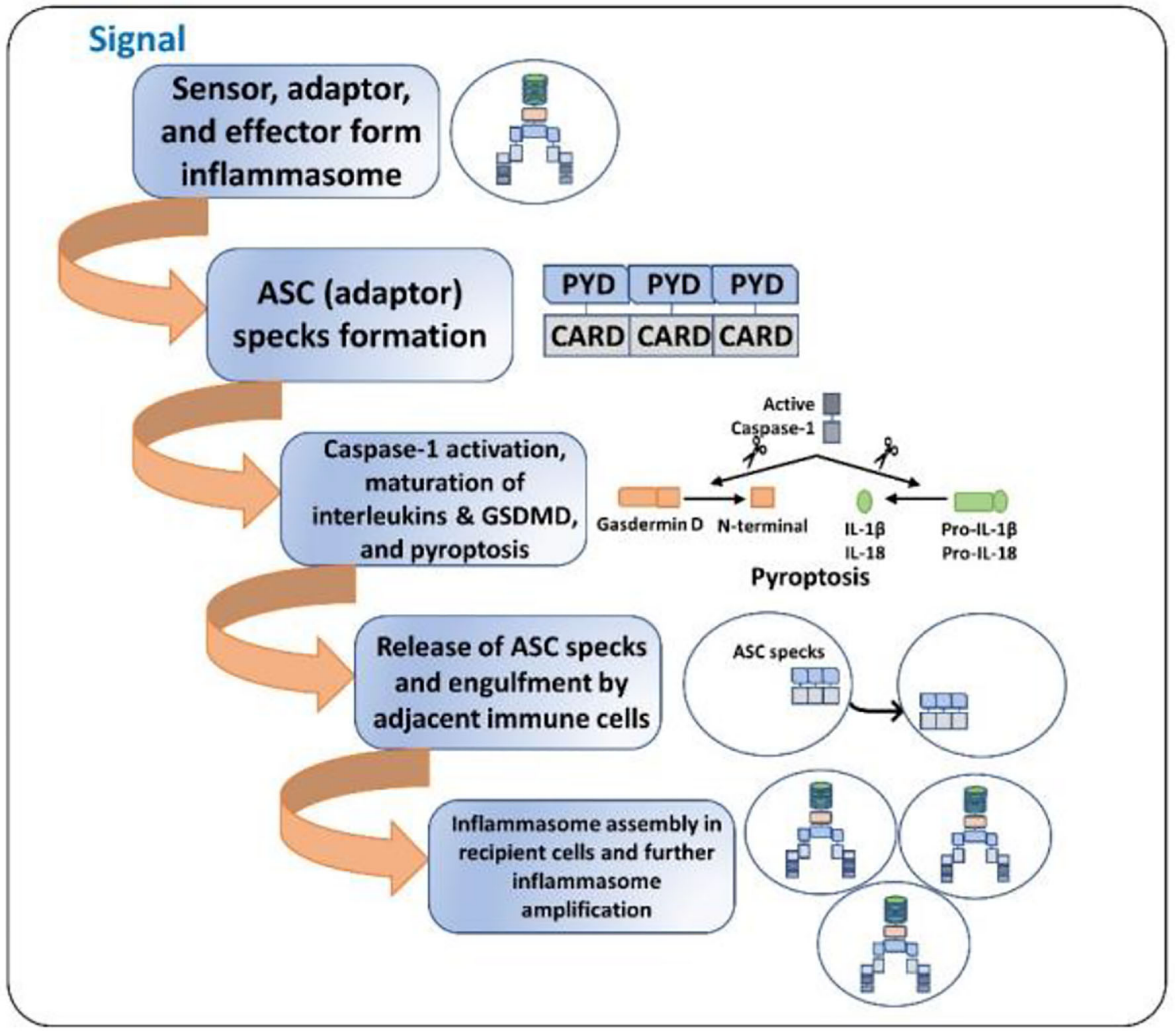
ASC specks and inflammasome activation amplification. 1. Initial signal (bacterial toxins, PAMPs, DAMPs, ds-DNA, etc.) leads to the assembly of the inflammasome. 2. ASC specks (oligomers) are formed such that the sensor, the adaptor, and the caspase-1 are always present at increasing concentrations. 3. The cell undergoes pyroptosis, resulting in cell lysis and the release of inflammatory cytokines and ASC specks. 4. The released ASC specks can be engulfed by adjacent immune cells, leading to the transduction of the upstream signal from one cell to the other. 5. The process of inflammasome assembly, activation, and pyroptosis repeats in the recipient immune cells, leading to amplification of inflammasome activation.

Inflammasome amplification is regulated by ASC *via* three mechanisms: first, sensors nucleate ASC, forming oligomers and ASC nucleates caspase-1 such that the sensor, adaptor, and enzyme are always present at cumulative concentrations; second, cytokines formed by caspase-1 infiltrate immune cells, lowering their activation; third, “ASC specks” released after pyroptosis can be engulfed by neighboring cells, forming an inflammasome in recipient cells (70, 71). These ASC specks are formed by phosphorylated-ASC and are crucial to inflammasome activity (72) (**Figure 2**). After the oligomerization of sensor proteins, inactive zymogen caspase-1 attaches itself to the scaffold and is activated by self-proteolysis into an active enzyme. Mouse caspase-1, -11, and -12 and human caspase-1, -4, and -5 are pro-inflammatory (73). Once activated, caspase-1 cleaves pro-IL-1β and pro-IL-18 and initiates their secretion, leading to an inflammatory form of cell death, pyroptosis. Pyroptosis consists of a formation of cell-membrane pores followed by cell swelling, osmotic lysis, and the release of intracellular debris (70, 74). Inflammatory caspase–dependent pyroptosis is carried out by the protein gasdermin D (GSDMD). Caspase-1, -4, -5, and -11 recognize and cleave to the same site in GSDMD, releasing its N-terminus, which signifies the autoinhibitory function of the C-terminus (75, 76). The N-terminus is the active form of GSDMD that forms pores, causing pyroptosis. Inflammasomes with caspase-1 as an effector are termed “canonical inflammasomes,” and caspase-11-mediated inflammasome activation is termed “non-canonical” (77). Nonetheless, activating the inflammasome works on an all-or-nothing principle (78), and multiple inflammasome sensors can orchestrate an inflammation response against a single pathogen inside a single host cell (79). In this review, we briefly discuss the most widely described inflammasomes in the literature (**Figure 1**).

### The NLRP1

It is the first PRR to be discovered that forms an inflammasome scaffold to mediate caspase-1–induced pyroptosis (80). Mice have three orthologs of NLRP1 (NLRP1a–c), whereas humans have a single gene coding for NLRP1. Murine orthologs have the CARD domain and lack the PYD domain, thus recruiting caspase-1 without ASC. In contrast, human NLRP1 has the N-terminal PYD domain, and NLRP1 is vital during host defense against anthrax (81). Mice lacking NLRP1 activation were more prone to the toxin due to aberrant host defense (82). In humans, macrophages induce apoptosis, not pyroptosis, in response to anthrax due to the absence of the *Nlrp1b* gene. Hence, NLRP1 is activated *via* binding to muramyl dipeptide (MDP) in the presence of the MDP sensor in humans (83). Single-nucleotide polymorphisms in the *Nlrp1* gene in humans are also associated with congenital toxoplasmosis, proving their importance in *Toxoplasma* infection as well (70).

### The NLRP3

The most widely studied inflammasome with respect to several human disorders is formed by NLRP3. It contains three domains—LRR, NACHT, and PYD—to bind ASC. The formation of the NLRP3 inflammasome is extensively studied in macrophages, where it is a two-step process: priming and activation (23, 84). PAMPs/DAMPs and cytokines induce non-transcriptional priming *via* post-translational changes (e.g., deubiquitination) and nuclear factor kappa B (NF-κB) activation *via* TLR4-induced transcriptional priming, leading to higher NLRP3 expression. The phosphorylation of ASC is also mandatory for NLRP3 scaffold formation. Non-transcriptional priming lasts a short (10–30 min) to intermediate time (30 min–1 h), while transcriptional priming lasts longer (>3 h) (23). TLR-mediated MyD88/IL-1 receptor-associated kinase 1 (IRAK1) regulates non-transcriptional NLRP3 priming, where NLRP3 expression does not change but priming is enough to secrete cleaved caspase-1 (85). A second signal is required for the activation step of NLRP3 and the formation of the NLRP3 signaling complex. A variety of “second signals” activate NLRP3, including potassium (K^+^) efflux, cathepsin release from lysosomal rupture, mitochondrial ROS and DNA, cardiolipin, calcium signaling, Na^+^, and Cl^-^ efflux, among others (23, 84). A newly identified NLRP3 binding partner, NEK7 (NIMA-related kinase 7), forms a NLRP3 inflammasome after K^+^ efflux (86). Many particulate substances, such as amyloid β (Aβ) (87), silica, alum, monosodium urate, etc., can cause lysosomal destabilization to activate NLRP3 inflammasome (88). Autophagy and related proteins inhibit the mitochondrial DNA release into cytosol, regulating NLRP3 activation (89). The dysregulation of the NLRP3 inflammasome is involved in the pathogenesis of a variety of human disorders, and a better understanding of NLRP3 activation would help identify drug targets for NLRP3-related diseases.

### The NLRC4

NLRC4 [also called ice protease-activating factor (IPAF)] contains the CARD domain to directly recruit caspase-1 to the inflammasome. The ASC is not needed for NLRC4-mediated pyroptosis but essential for amplifying the response and IL-1β release (10, 90). NLRC4 requires NLR-family apoptosis inhibitory proteins (NAIPs) to interact with bacterial pathogens upstream for its activation. NAIPs and their binding to NLRC4 have been reviewed in-depth elsewhere (91). After NAIPs bind to bacteria, NLRC4-NAIP-complexes are formed, leading to their activation. Besides pyroptosis, NLRC4 causes an actin polymerization response against *Salmonella*, highlighting the non-conventional role of NLRC4 inflammasome activation (92).

### The AIM2

It is a cytosolic DNA sensor from the ALR family characterized by a hematopoietic interferon-inducible nuclear protein with a 200-amino-acid (HIN-200) domain. AIM2 is activated by binding to double-stranded DNA (dsDNA) of a minimum 250–300 bp in a non-sequence-specific manner *via* the HIN200 domain (93). In the absence of cytosolic dsDNA, HIN200 binds to AIM2 PYD as an autoinhibition. The AIM2 PYD domain is displaced after dsDNA binding, forming the PYD-PYD interaction of ASC (84). The AIM2 inflammasome plays an incomparable role in host defense against bacteria, *Listeria*, and DNA viruses, as the impaired secretion of cytokines has been observed in macrophages lacking AIM2 (94). Interestingly, a recent report documented the requirement of AIM2 inflammasome surveillance of DNA damage for normal brain maturation and function. AIM2 contributes to the cell death of genetically compromised CNS cells and shapes overall behavior in mice (95). The dysfunction of AIM2 is linked to various human conditions.

### The Non-Canonical Inflammasome

Recent advances have identified a complex innate immune response phenomenon in which inflammasomes cleave caspase-11 in mice, termed “non-canonical inflammasome activation” (9). However, caspase-11 activation itself may stimulate the caspase-1–mediated release of canonical interleukins, as shown by the inhibition of the secretion of IL-1β and IL-18 under lipopolysaccharide (LPS) treatment in caspase-11-knockout mice (9). Additionally, caspase-11 is required for host defense against gram-negative bacterial infections since it detects specific acylated lipid A present in the LPS of this bacterial population (96). Caspase-11 is not activated in gram-positive bacterial infections, and caspase-11 activation results in the secretion of IL-1α and specific high-mobility group box-1 (HMGB1), triggering direct pyroptosis (77). TLR4 activation by LPS as a priming step is also dispensable in non-canonical signaling transduction, as HMGB1 promotes TLR4 signaling (97). It has been predicted that a caspase-11-bound pannexin-1–dependent decrease of intracellular K^+^ might activate the NLRP3 inflammasome (70), but further research is needed to understand whether the drop in intracellular K^+^ is sufficient to activate inflammasomes and/or whether other possible mechanisms are involved.

## Inflammasomes in Chronic Inflammatory Disorders

Inflammasomes are critical regulators of theoretically all chronic inflammatory disorders. Irregular signaling by inflammasomes inflicts perturbations on innate immune cells, eventually affecting adaptive immunity and being involved in the pathogenesis of acute and chronic inflammatory disorders. We discuss a few representative data that link various inflammasomes and the development of inflammatory diseases (**Table 1**).

**Table 1:** Role of major Inflammasomes in the representative chronic inflammatory disorders along with known cannabinoid effect.

Disorder	Inflammasomes	Pathophysiology	Genetic variations involved?	Possible reported effect of cannabis
Addison’s disease	NLRP1	Increased risk of autoimmunity	Yes (98)	A case report of Cannabis use disorder contributing to Addison’s (99)
Skin inflammation and cancer	NLRP1	Highly expressed in the skin; mutations in NLRP1 cause its self-oligomerization and atypical activation leading to various skin inflammatory conditions (100, 101)	Yes (100)	Anti-inflammatory, antipruritic, anti-aging, and anti-cancerous properties of cannabinoids along with mechanisms reviewed in details (102)
	NLRP3	NLRP3-dependent production of IL-1β may promote skin cancers	Yes (103, 104)	
	AIM2	AIM2 upregulation in acute and chronic skin inflammatory conditions and cancer (105, 106)	No	
Inflammatory bowel disease (IBD)	NLRP1	NLRP1 decreases the growth of beneficial gut bacteria promoting IBD (107)	No	Antioxidant and anti-inflammatory effects of cannabinoids on IBD reviewed in details (108)
	NLRP3	NLRP3 activation promotes IBD and not crucial for intestinal barrier maintenance (109)	Yes (110)	
	AIM2	AIM2 is an crucial regulator of intestinal inflammation *via* the IL-18/IL-22/STAT3 pathway (111)	No	
Systemic lupus erythematosus (SLE)	NLRP1	Upregulation of NLRP1 gene leading to higher IL-1β levels in SLE patients (112)	Yes (113)	Cannabidiol is not beneficial in the murine model of SLE (114), however, ajulemic acid (selective CB2 agonist) is highly beneficial; undergoing clinical trials (115).
	NLRP3	NLRP3 activation is involved in the differentiation of Th17 cells SLE mice (116)	Yes (112)	
	AIM2	AIM2 acts as na apoptotic DNA sensor in SLE causing macrophage activation (117)	No	
Type-1 diabetes (T1D)	NLRP1	Protective and detrimental role of NLRP1 variants depending on different ethnic population (118, 119)	Yes (113)	Increased risk of diabetic ketoacidosis (DKA) in type-1 diabetics who are moderate cannabis users (120) but cannabidiol treatment improves depression- and anxiety-like behavior in experimental type-1 diabetes in mice (121)
	NLRP3	NLRP3 is crucial for the expression of the chemokine receptors in T-cells regulating chemotaxis of immune cells in T1D mice (122)	Yes (123)	
	AIM2	AIM2 protects against T1D by reducing pancreatic pro-inflammatory response *via* IL-18 (124)	No	
Type-2 diabetes (T2D)	NLRP3	NLRP3-mediated IL-1β and IL-18 release and pyroptosis worsen insulin resistance and the progression of T2D, reviewed here (125). The activation of NLRP3 is upregulated in T2D patients (126)	Yes (127)	Chronic cannabis use was associated with visceral adiposity and insulin resistance in the adipose tissue (128), however, lifetime marijuana use showed lower insulin resistance in obese but not in non-obese adults (129).
	AIM2	Cell-free mitochondrial DNA activates AIM2 leading to Il-1β and IL-18-mediated inflammation in T2D patients (130) and AIM2 inhibition improved cardiac function in a diabetic rat model by blocking caspase-1 activity (131)	No	
	NLRC4	NLRC4 is a major contributor of IL-1β release in renal tissues contributing to the diabetic nephropathy (132)	Yes (133)	
Rheumatoid Arthritis (RA)	NLRP1	Inhibition of NLRP1 in arthritis model of mice significantly inhibited synovial inflammation (134)	Yes (98)	Cannabinoids are helpful in reducing pain and inflammation with RA *via* different mechanisms of action, reviewed here (135).
	NLRP3	Inhibition of NLRP3 in murine model of arthritis reduced the production of interleukin IL-1β and reduced inflammation of joints (136) and human patients with active RA showed higher expression and activation of NLRP3 (137)	Yes (137)	
	AIM2	Self-DNA sensed by AIM2 drives autoinflammation in mice with chronic polyarthritis mimicking RA in humans (138)	No	
Alzheimer’s disease (AD)	NLRP1	NLRP1 is involved in the neuroinflammation *via* IL-1β and IL-18-dependent neuronal pyroptosis along with NLRP1–caspase1–caspase6-mediated axonal degeneration and neuroinflammation leading to neuronal death (139). AD patients showed higher NLRP1 activation as well (140).	Yes (141)	Various studies, reviewed here (142), found limited evidence of the effectiveness of medical cannabis in neuropsychiatric symptoms associated with dementia. A well-structured randomized controlled trial (RCT) is needed to prove the clinical efficacy of medical cannabis in AD. However, cannabidiol, *via* multiple cannabinoid receptor independent mechanisms showed a positive impact on the progression of AD (143).
	NLRP3	NLRP3 is upregulated in an animal model of AD causing IFN1β production by microglia and inhibition of NLRP3 reduced the deposition of amyloid-β (140). AD patients exhibited NLRP3 inflammasome assembly and activation with high amounts of IL-1β and IL-18 (140).	Yes (144)	
	AIM2	Increased cytosolic DNA in traumatic brain injury detected by immune cells to activate AIM2 inflammasome and IL-1β and IL-18-dependent neuronal pyroptosis contributing to neurodegeneration in the pathogenesis of AD (145).	No	
	NLRC4	NLRC4 inflammasome, *via* IL-1β and IL-18, contributes to memory impairment and neuroinflammation in a rat model of Alzheimer-like disease (146).	No	
Parkinson’s disease (PD)	NLRP1	NLRP1 has been indirectly linked to PD by contributing to neuroinflammation and axonal degeneration *via* the caspase-1-caspase-6-mediated IL-1β pathway (147).	No	A systematic review found insufficient evidence to recommend the use of medical cannabinoids for motor symptoms in PD (148). A well-designed RCT is needed, however, cannabidiol has shown great potential as a prototype for drug development for PD (143).
	NLRP3	Several studies implicate a pathogenic role of NLRP3 in PD *via* IL-1β and IL-18-dependent pyroptosis. α-Synuclein activates TLR2 and TLR4-mediated NLRP3 inflammasome assembly and caspase-1 maturation both (149).	Rare NLRP3 polymorphism decreased the risk of PD (150)	
	AIM2	AIM2 inflammasome activity was augmented by inhibition of Parkinson’s disease-associated mitochondrial serine protease (151).	No	
	NLRC4	NLRC4 is crucial in regulating inflammation in aging (Inflammaging) which contributes to the development of neurodegenerative diseases like PD (152).	No	
Cardiovascular disorders (CVDs)	NLRP1	NLRP1 gene expression was found to be significantly higher in the patients with aortic occlusive disease (AOD) (153) and coronary stenosis (154) suggesting its importance in the development of atherosclerosis.	Yes (155)	Although marijuana use has been positively correlated with the increased risk of CVDs (156), several studies suggested the cardioprotective role of cannabidiol (157); suggesting a need for further research.
	NLRP3	NLRP3 has been implicated in multiple CVDs and inhibition of NLRP3 holds great potential for treating such disorders (109).	Yes, coronary artery disease (158)	
	AIM2	AIM2 hyper-activation is reported in a variety of CVDs including myocardial infarction (159) and atherosclerosis (160)	No	
	NLRC4	NLRC4 is involved in the pathophysiology of atherosclerosis (154) and myocardial infarction (159).	Yes (161)	
Cancers	NLR family	NLRP1, NLRP3, NLRC4, NLRP6, NLRP7, and NLRP12 have mixed roles in the pathogenesis of a variety of cancers as reviewed here in details (162, 163).	Yes (163)	Medical cannabis could be prescribed for nausea and vomiting after chemotherapy (14) but there is a weak evidence for their clinical efficacy in the management of cancer pain and other symptoms (164). RCTs are needed, however, non-THC cannabinoids show promising anti-cancerous actions (165).
	AIM2	Upregulation of AIM2 in oral, cervical, and lung cancer and downregulation in colorectal and small bowel cancer (166)	Yes (167)	

### Role of NLRP1

Genomic studies of NLRP1 have identified mutations associated with autoinflammatory diseases in humans, including systemic sclerosis, Crohn’s disease, Addison’s disease, rheumatoid arthritis, type-1 diabetes, and vitiligo (70, 98). NLRP1 is the most highly expressed inflammasome in human skin, and gain-of-function NLRP1 mutations cause chronic skin inflammation and skin cancer. These mutations result in the higher self-oligomerization of NLRP1, disrupting the PYD-LRR interaction crucial in keeping NLRP1 dormant under physiological situations (100). Genetic variations in NLRP1 are also positively associated with a higher susceptibility to psoriasis (101). Interestingly, higher NLRP1 expression is correlated to dry skin–induced chronic itch in a sex- and age-dependent manner in mice (168). NLRP1 has recently been implicated in the pathophysiology of IBD by restricting the beneficial butyrate-producing *Clostridiales* in the gut of the dextran sulphate sodium (DSS)-induced colitis murine model of IBD (107). Single-nucleotide polymorphisms in NLRP1 are associated with an increased risk of developing type-1 diabetes and systemic lupus erythematosus (SLE) in Brazilian population cohorts (113). Patients with aortic occlusive disease (AOD) have exhibited higher mRNA expression of NLRP1 than healthy individuals (153).

### Role of NLRP3

NLRP3 has been considered the gold standard of inflammasome signaling, as many NLRP3 inhibitors are under investigation in clinical trials for coronary artery disease (169) and gout (170); more than 50 clinical studies are currently underway to elucidate the role of NLRP3 in various diseases. Cryopyrin‐associated periodic syndrome (CAPS) is a well-documented autosomal‐dominant autoinflammatory disorder caused by gain-of-function mutations in NLRP3 in pediatric patients that lead to increased plasma IL-1β levels (171, 172). An enormous amount of data shows that NLRP3 is involved in the pathophysiology of Alzheimer’s, stroke and cardiovascular diseases, asthma, gout, IBD, non-alcoholic fatty liver disease, non-alcoholic steatohepatitis, multiple sclerosis, rheumatoid arthritis, myelodysplastic syndrome, obesity-induced inflammation or insulin resistance, type-1 diabetes, oxalate-induced nephropathy, graft-versus-host disease, and silicosis (109). Inflammaging is a condition marked by higher-than-normal levels of inflammatory markers in the blood, indicating a high risk of frailty. A major mechanism of inflammaging is abnormal NLRP3 inflammasome activation (173). In type-2 diabetes patients, NLRP3 inflammasome activation is higher in myeloid cells, and NLRP3 inflammasome inhibitors might be clinically useful in treating ischemic stroke concomitant with diabetes (126, 174). The activation of NLRP3 is involved in renal disorders, such as chronic kidney disease (CKD), diabetic nephropathy (DN), and acute kidney injury (AKI), by both canonical and non-canonical pathways (175). The NLR family (NLRP1, NLRP3, NLRC4, NLRP6, and NLRP12) and their binding partners have mixed roles in the pathogenesis of a variety of cancers (162). The serum levels of alpha-synuclein (α-synuclein) and caspase-1 are lower in Parkinson’s disease (PD) patients than in healthy individuals. Cleaved α-synuclein from caspase-1 enzymatic activity can form aggregates (176). Lower levels of caspase-1 and α-synuclein in PD patients’ serum are indicative of cell aggregate formation by both. Additionally, α-synuclein itself can activate the NLRP3 inflammasome, raising cytokine levels in PD patients (177). The field of targeting NLRP3 is continuously evolving and holds immense potential for the future of anti-inflammatory drug therapy.

### Role of NLRC4

Mutations in NLRC4 are associated with various autoimmune disorders. Three gain-of-function mutations (V341A, T337S, and H443P) in humans are linked to constitutive NLRC4 activation with recurrent macrophage activation syndrome (178, 179). NLRC4 activation primarily in neutrophils is enough to induce severe systemic autoinflammatory disease (180). Significant upregulation in mRNA and the protein levels of NLRC4 and NLRP3 has been found in urinary tract–infected female patients (181). Lastly, aberrant activation of NLRC4 is evident in non-alcoholic fatty liver disease (182), memory impairment in Alzheimer-like disease (146), myocardial infarction (159), and coronary stenosis (154). A genome-wide association study discovered that genetic variations in NLRC4 play vital roles in determining IL-18 levels in acute coronary syndrome patients (161).

### Role of AIM2

Circulating cell-free mitochondrial DNA (ccf-mtDNA) has been detected in the serum and plasma samples of type-2 diabetes patients. Research has shown that ccf-mtDNA-mediated AIM2 inflammation activation might be one of the contributing mechanisms of chronic inflammation in diabetic patients (130). AIM2 inflammasome is hyper-activated in type-2 diabetic mice with myocardial infarction (159). Blocking AIM2 expression improves cardiac function in a streptozotocin-induced diabetic rat model by preventing caspase-1–mediated cell signaling (131). Interestingly, AIM2 expression is higher in the kidney sections of patients with DN or hypertensive sclerosis than in healthy volunteers (183). The kidneys of mice with CKD have exhibited a higher expression of AIM2 mRNA, whereas AIM2-knockout mice kidneys have shown decreased maturation of IL-1β and IL-18 (184). AIM2 expression is positively associated with the severity of SLE in human patients and mice. Inhibition of the apoptotic DNA-induced macrophagic AIM2 activation is a key in AIM2 gene silencing–ameliorated SLE symptoms in mice (117). Furthermore, AIM2 expression is upregulated in oral, cervical, and lung cancer and downregulated in colorectal and small bowel cancer (166). AIM2 expression is protective in rheumatoid arthritis patients, leading to higher IL-1β release in the absence of AIM2 (185).

## Cannabinoids, Inflammasomes, and Human Diseases

So far, we have discussed the anti-inflammatory potential of various cannabinoids and the close association of inflammasomes with chronic inflammatory disorders. Several studies in the last few decades have suggested the potential of cannabinoids to modulate the inflammasome pathway. Below, we discuss research publications that established a mechanistic link between cannabinoids and inflammasome-related human diseases (**Tables 2** and **3**).

**Table 2:** Effects of cannabinoids on inflammasome proteins.

Cannabinoids	Inflammasome signaling	Effect observed	*In vitro* and/or *in vivo* model	Possible mechanism of action	Ref
Δ^9^-THC	IL-1β	Potentiation of Δ^9^-THC-induced catalepsy by IL-1β	Female BALB/c mice	Unknown/not established	(186)
	Caspase-1	Induction of apoptosis	Cultured murine immune cells	Modulation of caspase activity	(187)
	pro-IL-1β and caspase-1	Inhibition of monocyte/astrocyte interactions	Human astrocyte/monocyte co-culture	CB2 activation induced autophagy	(188)
	IL-1β	Reduction of inflammatory cytokine IL-1β	Monocytes from patients with inflammatory arthritis	Not studied	(189)
	IL-1β	Inhibition of IL-1β and NF-κB after LPS stimulation	Human osteosarcoma cells MG-63	CB2 activation	(190)
	IL-1β	Reduction of IL-1β and anti-inflammatory activity	Adjuvant-induced arthritis in rats	Not established	(191)
Δ^8^-THC	Caspase-1	Cytotoxicity	Mouse macrophage J774-1 cells	CB2 receptor and p38 MAPK dependent	(192)
Δ^9^-THCV	IL-1β	Inhibition of LPS-induced IL-1β release	Murine peritoneal macrophages	CB2 activation and not CB1	(193)
CBD	Caspase-1, and IL-18	Reduction of mRNA and protein levels of caspase-1 and IL-18	Human gingival mesenchymal stem cells	Inhibition of NF-κB and NLRP3	(194)
	Caspase-1, ASC, and IL-1β	Protection of liver from non-alcoholic steatohepatitis	High-fat, high-cholesterol (HFC) diet C57BL/6J mice and RAW264.7 murine macrophages	Inhibition of NF-κB and NLRP3 pathway	(195)
	IL-1β	Inhibition of neuroinflammation *via* reducing IL-1β	LPS-stimulated murine microglia	Independent of CB1 and CB2	(196, 197)
	IL-1β	Anti-inflammatory action in Abeta evoked neuro-inflammation	Mice injected with human Abeta (1–42) peptide	Not studied	(198)
	IL-1β	Anti-inflammatory action *via* reducing IL-1β levels	Murine model of colitis	Unknown	(199)
	IL-1β	Anti-inflammatory role by the inhibition of IL-1β	Viral model of multiple sclerosis in female SJL/J mice	Not established	(200)
Ajulemic acid	IL-1β	Inhibition of inflammation by reducing IL-1β levels	Monocytes from patients with inflammatory arthritis	Selective CB2 receptor agonism	(189)
JD5037	IL-1β, IL-18, caspase-1	Inhibition of IL-1β, IL-18 release and caspase-1 activity promoting normoglycemia	Zucker diabetic fatty (ZDF) rats	Peripheral CB1 receptor inverse agonism leading to NLRP3 inflammasome deactivation	(201)
HU‐308	IL-1β, caspase-1	Anti-inflammatory effect by decreasing IL-1β production and caspase-1 activity	Mouse model of experimental autoimmune encephalomyelitis	Selective CB2 blockade leading to autophagy-mediated inhibition of NLRP3	(202)
AJ5012	IL-1β, caspase-1	Suppression of adipose tissue inflammation by inhibition of IL-1β levels and caspase-1 activity	Diet-induced obese (DIO) and leptin receptor–deficient C57BL/6J mice	Peripheral CB1 receptor antagonism-mediated inhibition of NLRP3 pathway	(203)
Rimonabant	IL-1β	Reduction of inflammation in atherosclerosis by decreasing IL-1β	LDL receptor–deficient mice	Selective CB1 receptor antagonism	(204)
JWH-133 and HU-308	IL-1β and caspase-1	Decreased serum IL-1β and IL-1β mRNA and lower caspase-1 activity-anti-inflammatory and improved cardiac function	Surgically induced myocardial infarction (MI) mice	Selective CB2 receptor agonism-stimulated NLRP3 suppression	(205)
AM1241	IL-1β and caspase-1	Anti-inflammatory potential *via* reduced IL-1β and matured caspase-1 protein expressions	Rat macrophagic NR8383 cells treated with complete Freund’s adjuvant (CFA)	Selective CB2 agonism-mediated NLRP3 suppression	(206)
HU-308	IL-1β and caspase-1	Anti-inflammatory actions *via* reduced levels of IL-1β and matured caspase-1	LPS/DSS-induced *in vitro* colitis model of freshly isolated murine macrophages	Selective CB2 blockade leading to autophagy-mediated inhibition of NLRP3	(207)

**Table 3:** Effects of cannabinoids on inflammasomes.

Cannabinoids	Inflammasome	Effect observed	*In vitro* and/or *in vivo* model	Possible mechanism of action	Ref
CBD	NLRP3	Inhibition of NLRP3 mRNA and protein	Human gingival mesenchymal stem cells	Possibly *via* inhibiting NF-κB-induced priming	(194)
	NLRP3	Protection of liver from non-alcoholic steatohepatitis	High-fat, high-cholesterol (HFC) diet C57BL/6J mice and RAW264.7 murine cells	Inhibition of NF-κB- mediated priming step	(195)
	NLRP3	Cytoprotection against UV rays *via* inhibition of NLRP3	Human keratinocytes treated with UV-A and UV-B rays	Reduction of NF-κB levels by inhibiting Nrf-2 and NF-κB interaction	(208)
	NLRP3	Anti-inflammatory action *via* impeding NLRP3	LPS-nigericin-stimulated human THP-1 monocytes	Decreased potassium efflux by modulating P2X7 receptors	(209)
JD5037	NLRP3	Normoglycemia and improved type-2 diabetes profile by NLRP3 inhibition	Zucker diabetic fatty (ZDF) rats	Peripheral CB1 receptor inverse agonism	(201)
HU‐308	NLRP3	The anti-inflammatory effect in human multiple sclerosis model *via* NLRP3 blockade	Mouse model of experimental autoimmune encephalomyelitis	Selective CB2 receptor activation promotes autophagy leading to inhibition of NLRP3	(202)
AJ5012	NLRP3	Suppression of adipose tissue inflammation by NLRP3 inhibition	Diet-induced obese (DIO) and leptin receptor–deficient C57BL/6J mice	Peripheral CB1 receptor antagonism	(203)
JWH-133	NLRP3	Anti-inflammatory action *via* decreased priming and activation of NLRP3	Primary murine cardiomyocytes treated with oxygen-glucose deprivation	Selective CB2 receptor agonism	(205)
AM1241	NLRP3	Anti-inflammatory potential *via* NLRP3 assembly inhibition	Rat macrophagic NR8383 cells treated with complete Freund’s adjuvant (CFA)	Selective CB2 agonism	(206)
HU-308	NLRP3	Anti-inflammatory potential *via* NLRP3 inhibition	LPS/DSS-induced *in vitro* colitis model of freshly isolated murine macrophages	Selective CB2 receptor activation promotes autophagy leading to inhibition of NLRP3	(207)

### Tetrahydrocannabinol (THC) and Analogs

The first reports on the effect of Δ^9^ THC on IL-1β (186) and caspase-1 (187) were published in the 1990s. Δ^9^ THC has been found to reduce the levels of pro-IL-1β and inflammasome-induced caspase-1 activation in human astrocyte-monocyte co-culture *in vitro* (188). In these cells, Δ^9^ THC inhibited caspase-1 activity, as shown by a reduction in IL-1β levels at a concentration as low as 0.5 μM. The authors confirmed that the CB2R activation-mediated induction of autophagy was the best possible mechanism by which Δ^9^ THC was inhibiting inflammasome activation, as both Δ^9^ THC and JWH-015 (selective CB2 agonists) showed similar results (188). Δ^8^ THC, an isomer of Δ^9^ THC, was reported to induce cell death *via* a caspase-1–dependent pathway in mouse macrophages, activating CB2R followed by the activation of p38 MAPK (192). The effect of Δ^9^ THC on reducing IL-1β mRNA and protein levels was comparable to ajulemic acid, a novel CB2 agonist, in monocytes isolated from patients with inflammatory arthritis (189). In another study, Δ^9^ THC was able to reduce IL-1β and NF-κB levels *via* CB2R activation in a human osteosarcoma cell line after LPS stimulation (190). Δ^9^-tetrahydrocannabivarin (THCV) was also shown to inhibit IL-1β levels in LPS-challenged murine macrophages (193). Feeding Δ^9^ THC mixed with sesame oil orally to rats with chemically induced rheumatoid arthritis (RA) for 21 days significantly reduced IL-1β concentration to baseline, suggesting the possible inhibition of inflammasomes as a promising target in RA (191). Although Δ^9^ THC displayed a significant effect on the inflammasome pathway, direct action on inflammasome sensor proteins has not yet been reported. It has been pointed out that a Ca^2+^ channel, TRPV2, is activated by Δ^9^ THC in myeloid cells and that TRPV2 stimulation leads to NLRP3 inflammasome activation, but no studies have confirmed this (210).

### Cannabidiol (CBD)

The first report on the direct effect of CBD on the inflammasome came in 2016 from a group of Italian researchers (194). They treated human gingival mesenchymal stem cells (hGMSCs) for 24 h with CBD (5 μM) and performed gene expression analysis and immunocytochemistry. They discovered that CBD-treated hGMSCs suppressed NLRP3, caspase-1, and IL-18 at the gene and protein levels and inhibited NF-κB. As NF-κB is involved in the priming of the NLRP3 inflammasome, the CBD treatment–induced inactive state of the NLRP3 inflammasome in hGMSCs (194) suggested that CBD-treated gingival stem cells were more immunocompetent, avoiding the risk of inflammatory reactions and promoting survival. Mice fed with a high-fat, high-cholesterol diet (HFC) for 8 weeks showed significantly higher expressions of NLRP3 inflammasome pathway proteins (NLRP3, ASC, IL-1β, and caspase-1) in the liver; these proteins were significantly attenuated by simultaneous treatment with CBD (5 mg/kg/day for 8 weeks). Similarly, the phosphorylation of NF-κB was significantly reduced in the liver of CBD-treated HFC mice compared to the non-treated group, corroborating the role of NF-κB in priming the NLRP3 inflammasome. To further confirm the role of the inflammasome in liver inflammation, the authors studied the effect of CBD on an LPS + ATP treated mouse macrophage cell line, confirming with *in vivo* data that the expressions of NLRP3, ASC, IL-1β, NF-κB, and caspase-1 were lower in CBD-treated cells (195). Mouse microglial cells treated with LPS to simulate neuroinflammatory conditions exhibited a robust activation of pro-inflammatory cytokine repertoire, and CBD (1–10 μM) was able to suppress the secretion of IL-1β and inhibit the NF-κB signaling pathway (196). A similar reduction in the secretion of IL-1β by CBD (10 μM) was also reported by another independent report (197). In the *in vitro* skin inflammation model, human keratinocytes were treated with ultraviolet (UV) rays A and B (UVA and UVB) and treated with CBD (1 μM) for 24 h. CBD inhibited protein-protein interaction between nuclear factor erythroid 2-related factor 2 (Nrf2) and NF-κB in UVA- and UVB-treated skin cells. CBD increased NRF2 expression, leading to decreased ROS, which in turn may have partially suppressed NLRP3 inflammasome activation by reducing NF-κB levels (208). Aβ-induced neurotoxicity (198) and the severity of inflammatory colitis (199) were significantly suppressed by CBD treatment in mice partly due to inhibiting the expression and release of IL-1β. A significant reduction of IL-1β by CBD treatment in a murine viral model of multiple sclerosis was also shown, suggesting its role in combating inflammation in multiple sclerosis (200). Recently, CBD was shown to inhibit NLRP3 inflammasome by reducing K^+^ efflux by binding to the P2X7 receptor in human monocytes (209). Remarkably, the fact that only CBD (non-psychoactive), not THC (psychoactive), was found to inhibit NF-κB signaling (197), coupled with the direct proven inhibitory action of CBD on inflammasomes (195) and downstream proteins, indicates the incomparable potential of CBD as an inflammasome-inhibitory drug target.

### Synthetic Cannabinoids

Synthetic cannabinoids often act by modulating the action of endocannabinoids on CB1 and CB2 receptors to exert their anti-inflammatory effects. Endocannabinoids mediate insulin resistance by activating peripheral CB1R. Beta-cell failure in Zucker diabetic fatty (ZDF) rats was not associated with CB1R in beta cells; but rather, macrophages infiltrating the pancreas activated the NLRP3 inflammasome machinery. Macrophage-specific knockdown and/or peripheral blocking of CB1R *via* the non-CNS-penetrant CB1R inverse agonist JD5037 improved the pathology of type-2 diabetes and re-established normoglycemia by reducing the expression of cardinal NLRP3 inflammasome proteins (201). However, CB1R has detrimental effects on beta-cell function, and its activation promotes islet inflammation under pathological insults. Beta-cell-specific CB1R gene knockout increased insulin secretion and cAMP levels in islets. High-fat/high-sugar-induced inflammation was attenuated significantly by the absence of CB1R in beta cells *via* the reduction of ROS and suppression of NLRP3 inflammasome activation (211). Contrary to the protective role of CB2R in diabetic inflammation, in a mouse model of experimental autoimmune encephalomyelitis (EAE) (an experimental model for human multiple sclerosis), CB2R mRNA and NLRP3 protein expressions were significantly higher with unchanged CB1R mRNA expression (212). The authors did not study the protein expression of CB1R and CB2R in this report, but using the same multiple sclerosis model, another group of researchers showed an exacerbated NLRP3 inflammasome response in CB2R knockout mice and amelioration of the response in wild-type mice by selective CB2R activation by HU‐308 (202). It was discovered that CB2R activation by HU‐308 induces autophagy in mouse microglial cells, inhibiting NLRP3 activation. The discrepancy in the results may be due to the use of only female mice and not analyzing cannabinoid receptors at the protein level (212). An antagonist of peripheral CB1R, AJ5012, prevents adipose tissue inflammation in leptin receptor–deficient and diet-induced obese (DIO) mice by inhibiting NLRP3 inflammasome signaling. Reductions in the protein expressions of caspase-1, IL-1β, and NLRP3, along with caspase-1 activity, were noticed in the presence of AJ5012 in DIO mice. AEA-induced increases in the expressions of NLRP3, CB1R, and IL-1β in murine macrophage cells were also significantly attenuated by AJ5012 (203, 213). These data indicate the critical role of the NLRP3 inflammasome in adipose tissue inflammation *via* peripheral CB1R signaling. Rimonabant, a selective CB1R antagonist, inhibits the development of atherosclerosis in low-density lipoprotein (LDL) receptor–deficient mice, partially by reducing IL-1β–mediated pro-inflammatory gene expression (204). Myocardial infarction (MI) is a major cardiovascular event with a high mortality rate in post-MI heart failure. A selective CB2R agonist, JWH-133, improved heart function in a surgically induced MI mice model. JWH-133 treatment reduced serum IL-1β and IL-1β mRNA expressions even at a 1 mg/kg concentration. The administration of JWH-133 at a 10 mg/kg dose significantly diminished the priming and activation of the NLRP3 inflammasome, as shown by levels of inflammasome protein expression (205). Electroacupuncture (EA) attenuated inflammatory pain induced by complete Freund’s adjuvant (CFA) in rats by blocking NLRP3 activation in skin macrophages. This effect was largely attributed to CB2R, as CB2R-knockout mice exhibited a loss of EA effects on pain. In rat macrophagic cell lines, CFA- and LPS+ATP–induced NLRP3 activation was significantly inhibited by the selective CB2R agonist AM1241 (206). In freshly isolated mice macrophages, the CB2R selective agonist HU-308 significantly blocked the expression of NLRP3 inflammasome assembly proteins in an LPS/DSS-induced *in vitro* colitis model (207). Selective inhibitors of CB1R and activators of CB2R have shown tremendous potential in alleviating inflammatory disorders partly by affecting NLRP3 inflammasome assembly and activation.

Terpenoids and flavonoids are abundantly present in cannabis extracts, and both molecule groups exhibit anti-inflammatory activities. Many terpenoids and flavonoids are inflammasome inhibitors in general and are reviewed extensively elsewhere (214–217), a subject beyond the scope of this review.

## Relevance to The Coronavirus Disease 2019 (COVID-19) PANDEMIC

The ongoing COVID-19 outbreak is a global pandemic caused by a novel coronavirus named “severe acute respiratory syndrome coronavirus 2” (SARS-CoV-2). The angiotensin-converting enzyme 2 (ACE2), a metallopeptidase, is a known functional receptor for coronaviruses, and their surface spike glycoproteins (S) bind physically to ACE2 (218–220). A high expression of ACE2 is correlated to innate and acquired immune response, cytokine secretion, and enhanced inflammatory response in COVID-19 patients. A clinical study in Wuhan pointed out that the levels of IL-1β, IL-10, and IL-8 were significantly higher in critically ill patients with SARS-CoV-2 infection, indicating a cytokine-mediated inflammatory response (221). Recently, it was established that the SARS-CoV open reading frame (ORF)-8b activates the NLRP3 inflammasome affecting innate immunity (222) and SARS-CoV viroporin 3a protein independently activates NLRP3 inflammasome in macrophages isolated from adult mice (223). Very recently published reviews strongly suggest that SARS-CoV-2 could directly activate NLRP3 inflammasome and NLRP3 activation could be a potential drug target in the treatment of COVID-19 (224, 225). In our laboratory, we established several novel high-CBD *C. sativa* extracts that significantly inhibited the expression of ACE2, entry point of the SARS-CoV-2 (226). The reason for choosing high-CBD cannabis extracts was to avoid psychoactive side-effects of Δ^9^-THC and to avoid CB1 agonism-mediated pathologic changes observed in the pulmonary tissues (227). Therefore, with the established evidence suggesting the role of cannabinoids as key regulators of inflammasome signaling, the vital cannabinoid moieties (CBD and THC) might be beneficial in alleviating the inflammatory aspects of COVID-19 by blocking inflammasome signaling.

## Conclusions and Clinical Relevance

Inflammation is a crucial phenomenon in understanding the pathophysiology of a variety of inflammatory disorders, and many anti-inflammatory antibodies are important treatment options for moderate-severe inflammatory diseases. The contribution of inflammasomes in the regulation of human disorders has been emphasized in research within the last few decades. However, no inflammasome-targeted therapy is currently approved for human use.

Cannabis has been shown to possess anti-inflammatory effects owing to its constituents, cannabinoids and terpenoids. New evidence is accumulating on the potential inhibitory action of cannabinoids on NLRP3 and other inflammasomes leading to their potent anti-inflammatory effects. On the other hand, cannabinoids with CB1 receptor agonist activity exhibit pro-inflammatory effects by inflammasome activation *via* CB1 agonism. The summarized reports here showed *in vitro* and *in vivo* data on cannabinoids modulating inflammasome activity and proving beneficial in reducing the pathogenicity of chronic inflammatory diseases. Cannabinoids also target crucial proteins involved in the inflammasome signaling, including NF-κB, IL-1β, etc.

**Figure 3:**
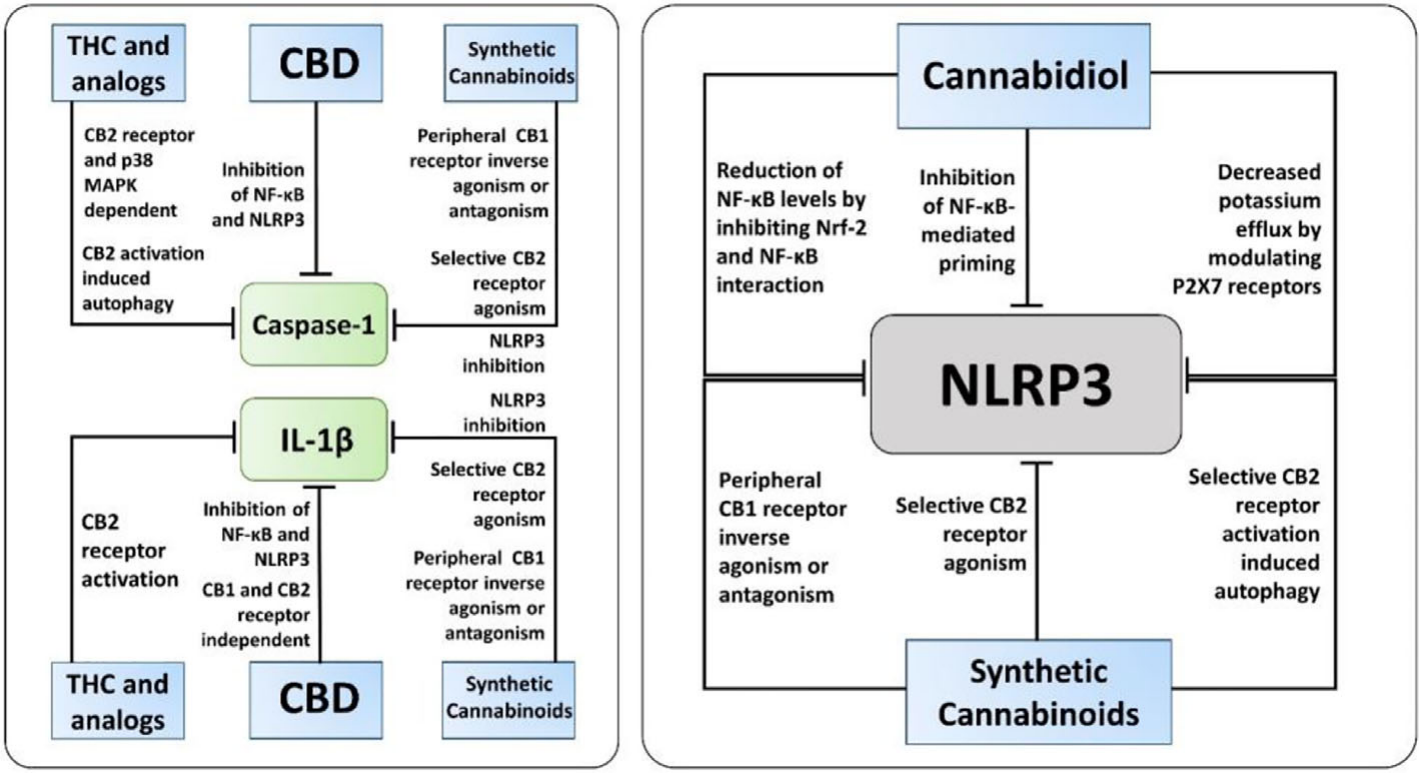
The known molecular pathways of inhibition of Caspase-1, IL-1β, and NLRP3 by cannabinoids. Along with NLRP3 activation, cannabidiol (CBD), synthetic cannabinoids, and Δ^9^-tetrahydrocannabinol (THC) and its analogs inhibit caspase-1, IL-1β, and IL-18 *via* different mechanisms independent of inflammasome activation. Notably, only CBD and synthetic cannabinoids, not THC and its analogs, have been reported to have a direct inhibitory action on NLRP3 activation.

The exact molecular mechanisms by which cannabinoids modulate inflammasome signaling have not been investigated completely, nevertheless, the current evidence supports their importance as promising therapeutic targets to regulate inflammasome signaling (**Figure 3**). Targeted inhibition of inflammasome by cannabinoids may prove beneficial over the global inhibition of cytokines, which increases the chances of infection as a side effect. Overall, cannabinoids hold a great promise as additional therapeutics to support the current treatment of chronic inflammatory diseases, along with COVID-19, however it should be weighed against pro-inflammatory actions mediated by CB1-agonism. Hence, cannabinoids with CB1 receptor antagonist and CB2 receptor agonist activity, for instance cannabidiol, should be considered for future research.

## Author Contributions

Conceptualization: SS. Writing, reviewing, and editing: SS, IK, and OK. All authors contributed to the article and approved the submitted version.

## Funding

Research was supported by the CIHR and MITACs grants to IK and OK.

## Conflict of Interest

IK and OK are involved with Pathway RX, a biotech start-up company focused on medical cannabis, albeit this start-up has no bearing on the materials summarized in the current review.

The remaining author declares that the research was conducted in the absence of any commercial or financial relationships that could be construed as a potential conflict of interest.
